# A generalizable machine learning framework for classifying DNA repair defects using ctDNA exomes

**DOI:** 10.1038/s41698-023-00366-z

**Published:** 2023-03-13

**Authors:** Elie J. Ritch, Cameron Herberts, Evan W. Warner, Sarah W. S. Ng, Edmond M. Kwan, Jack V. W. Bacon, Cecily Q. Bernales, Elena Schönlau, Nicolette M. Fonseca, Veda N. Giri, Corinne Maurice-Dror, Gillian Vandekerkhove, Steven J. M. Jones, Kim N. Chi, Alexander W. Wyatt

**Affiliations:** 1 Vancouver Prostate Centre, Department of Urologic Sciences, University of British Columbia, Vancouver, BC Canada; 2 Department of Medical Oncology, BC Cancer, Vancouver, BC Canada; 3 Yale School of Medicine and Yale Cancer Center, New Haven, CT USA; 4 Michael Smith Genome Sciences Centre, BC Cancer, Vancouver, BC Canada

**Keywords:** Prostate cancer, Computational biology and bioinformatics, Tumour biomarkers, Cancer genomics

## Abstract

Specific classes of DNA damage repair (DDR) defect can drive sensitivity to emerging therapies for metastatic prostate cancer. However, biomarker approaches based on DDR gene sequencing do not accurately predict DDR deficiency or treatment benefit. Somatic alteration signatures may identify DDR deficiency but historically require whole-genome sequencing of tumour tissue. We assembled whole-exome sequencing data for 155 high ctDNA fraction plasma cell-free DNA and matched leukocyte DNA samples from patients with metastatic prostate or bladder cancer. Labels for DDR gene alterations were established using deep targeted sequencing. Per sample mutation and copy number features were used to train XGBoost ensemble models. Naive somatic features and trinucleotide signatures were associated with specific DDR gene alterations but insufficient to resolve each class. Conversely, XGBoost-derived models showed strong performance including an area under the curve of 0.99, 0.99 and 1.00 for identifying BRCA2, CDK12, and mismatch repair deficiency in metastatic prostate cancer. Our machine learning approach re-classified several samples exhibiting genomic features inconsistent with original labels, identified a metastatic bladder cancer sample with a homozygous *BRCA2* copy loss, and outperformed an existing exome-based classifier for BRCA2 deficiency. We present DARC Sign (DnA Repair Classification SIGNatures); a public machine learning tool leveraging clinically-practical liquid biopsy specimens for simultaneously identifying multiple types of metastatic prostate cancer DDR deficiencies. We posit that it will be useful for understanding differential responses to DDR-directed therapies in ongoing clinical trials and may ultimately enable prospective identification of prostate cancers with phenotypic evidence of DDR deficiency.

## Introduction

Alterations in DNA damage repair (DDR) genes are common in metastatic castration-resistant prostate cancer (mCRPC)^1,2^. Deleterious germline and/or somatic mutations in homologous recombination repair (HRR) related genes including *BRCA2*, *ATM*, and *CDK12* are present in 15–20% of patients^1,3–5^. A further 3–5% exhibit alterations in mismatch repair (MMR) genes *MSH2, MSH6* or *MLH1*^3,6,7^. Collectively, these gene alterations play a critical role in patient management, directly influencing systemic therapy selection. Poly (ADP-ribose) polymerase (PARP) inhibitors are approved for HRR gene-mutated mCRPC^2^. Platinum chemotherapy also has activity in mCRPC with HRR gene defects^8–11^. MMR deficient (MMRd) mCRPC responds to immune checkpoint inhibition, and *CDK12* alterations have been linked to sensitivity to immunotherapy^5,6^. Unfortunately, even among biomarker-selected patients, clinical response rates to each class of treatment are sub-optimal^12–14^.

Since DDR is proficient in most mCRPC^4^, the utility of PARP inhibitors and other emerging therapies depends on accurate identification of vulnerable tumours^2^. The preferred clinical approach is to perform targeted sequencing across the exons of DDR genes in archival prostate biopsy tissue. However, gene alteration status from targeted sequencing is an incomplete predictor of DDR proficiency^15^. Firstly, targeted approaches may miss complex structural rearrangements, resulting in false negatives^7^. Secondly, evaluation of pathogenicity is imperfect, especially for missense mutations and non-*BRCA* genes^16^. Thirdly, biallelic loss can be difficult to discriminate from monoallelic loss^17^. Because most DDR genes are presumed haplosufficient, durability of response to targeted therapies is most strongly correlated with biallelic gene inactivation^5,18^. Finally, the clinical relevance of mutations in rarer DDR genes are unclear due to the anecdotal nature of any observed therapy response.

The most commonly altered DDR genes are associated with distinct patterns of genomic alterations. Defective *MSH2* drives microsatellite instability and high tumour mutational burden^7,19^. *CDK12*-altered mCRPC exhibits genome-wide focal tandem duplications^17,20^. *BRCA2* (though not *ATM*) defects are associated with mutational signatures of defective HRR, as in breast, ovarian, and pancreatic cancer^21,22^. In other cancers, innovative models have been developed to accurately identify defective HRR using mutational features from whole-genome sequencing^15,23,24^. However, different cancer types exhibit distinct mutational rates and processes, which influence model attributes and overall performance, especially in different clinical contexts and/or cancers not considered during model development^15,23^. Few models have been specifically developed for prostate cancer, which is characterised by widespread copy number alterations, complex structural rearrangements and comparatively low mutational burden, independent of DDR status^22,25^. No tools account for prostate cancer-specific features or can simultaneously identify *BRCA2* deficient (BRCA2d), *CDK12* deficient (CDK12d), and MMRd mCRPC from individual patient samples.

**Fig. 1:**
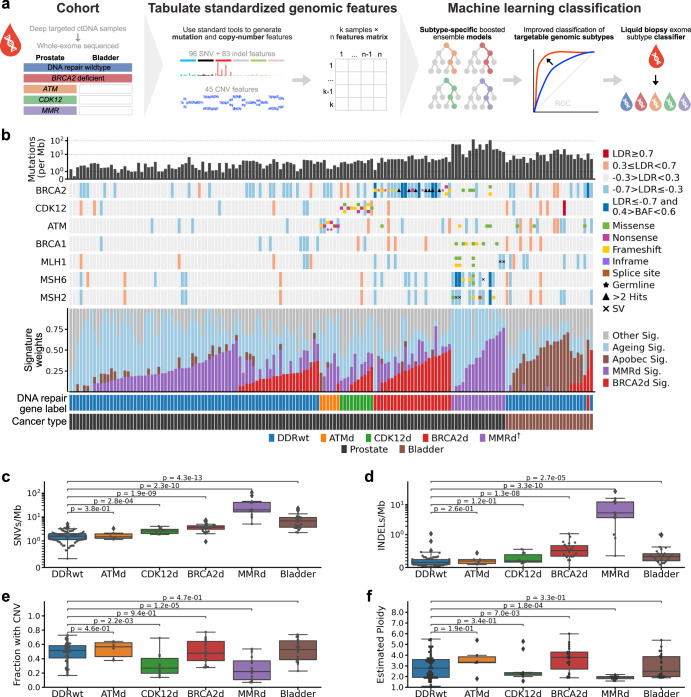
Dataset overview. **a** Graphical abstract depicting development of a machine learning classifier for identifying DNA damage repair defects in metastatic prostate and bladder cancers. Pre-evaluated clinical ctDNA samples from multiple sources were selected for whole-exome sequencing and used to train interpretable XGBoost models. **b** Oncoprint showing assigned DNA damage repair labels (assigned from prior deep targeted sequencing) and selected somatic features of the whole-exome sequencing cohort including signature weights and mutation counts. Log depth ratios (LDR) are the normalised average from targeted sequencing. **c**–**f** Comparison between naïve somatic features of all samples assigned to each DNA damage repair label, including the number of single nucleotide variants (SNVs) and indels, the proportion of the genome affected by a copy number variant (CNV) relative to base ploidy, and the overall genome ploidy. *P*-values are from Mann–Whitney *U* tests. For the box and whisker plots, the box encompasses the interquartile range, the midpoint of the box represents the median, and the whiskers extend 1.5× beyond the interquartile range. ^†^Note that the mismatch repair defective (MMRd) label reflects integrated results from ctDNA gene panel sequencing, whole-exome sequencing, intron sequencing as well as immunohistochemical staining of MSH2, MSH6, MLH1, and PMS2 in archival primary tissue.

Routine whole-genome sequencing of tumour tissue biopsy is clinically unfeasible in mCRPC^26,27^. However, plasma circulating tumour DNA (ctDNA) is abundant in a large proportion of clinically-progressing mCRPC^28^, enabling identification of genomic features including copy number changes and mutations^29^. We recently demonstrated that published trinucleotide signatures of defective MMR can be inferred from whole-exome sequencing (WES) of ctDNA^19^. Here, we exploit algorithmic advances in boosted ensemble models^30,31^ to develop DARC Sign (DnA Repair Classification Signature) (Fig. 1a): a set of models and accompanying software for classifying clinically-actionable DDR deficiencies in prostate cancer using ctDNA WES.

## Results

### Clinical plasma ctDNA samples subjected to whole-exome sequencing

To construct a labelled training dataset for generating DARC Sign classification models (Fig. 1a), we assembled WES data of plasma cell-free DNA and matched leukocyte DNA from patients with metastatic prostate cancer. WES data from patients with metastatic bladder cancer were included as a comparator (but were not used for model training). Samples and corresponding labels for DNA damage repair gene status were drawn from four published studies from our group employing the same experimental and computational methodology^17,19,28,32^. This data was supplemented here with WES data from additional samples harbouring DNA damage repair gene alterations identified via deep targeted sequencing. After quality control, our cohort consisted of 155 cell-free DNA samples with WES data, including 129 from metastatic prostate cancer and 26 from bladder cancer; median on-target unique read depth 164x (Supplementary Data 1, 2). To maximise generalizability of results, we applied Sequenza^33^, an established tool for tumour fraction (purity) estimation and identification of copy number states. The median Sequenza-assigned tumour fraction across our samples was 37% (Supplementary Data 1).

Our cohort included 23 metastatic prostate cancer samples with germline and/or somatic BRCA2d, 10 with CDK12d, 16 with MMRd, and 6 with *ATM* defects (ATMd) (Fig. 1b; Supplementary Data 1). The remaining 74 samples from patients with prostate cancer were labelled as DDR gene wild type (DDRwt) based on the absence of deleterious alterations in established DDR genes in targeted and whole-exome sequencing data. 25 of 26 bladder cancer samples were labelled as DDRwt, with one labelled as BRCA2d due to a deep copy number deletion of *BRCA2*. Mutation and copy number calls from WES data mirrored original labels from deep targeted sequencing (Fig. 1b; Supplementary Data 3, 4).

### Low specificity of conventional trinucleotide signature fitting

Decomposition of trinucleotide profiles and subsequent signature fitting can identify BRCA2d but is most suited to whole-genome sequencing data where there are large numbers of mutations. We evaluated COSMIC trinucleotide mutation signature weights^34^ across our WES dataset, combining weights for MMRd-associated signatures 6, 15, 20, and 26 (Fig. 1b). Sensitivity was high for detection of samples with MMRd (with minimum weight of 0.05, sensitivity = 0.93) and BRCA2d (signature 3; sensitivity = 0.91). However, specificity was low (BRCA2d = 0.69; MMRd = 0.21). The particularly low specificity for MMRd detection is likely attributable to the advanced age of patients with prostate cancer and the similarity between signatures associated with MMRd and ageing. There is no known trinucleotide signature for CDK12d (or ATMd). The poor discriminatory value of fitted trinucleotide signatures in isolation, together with the lack of a known mutational signature for CDK12d, highlights the need for incorporation of additional genomic features into DDR defect classification tools for WES data.

### Individual genomic features associate with distinct DNA damage repair gene defects but do not accurately classify prostate whole-exomes

Next we examined the burden of mutations and copy number alterations in each sample (Fig. 1c–f). As expected, both BRCA2d and MMRd samples showed significantly more single nucleotide variants (SNVs) and insertions and deletions (InDels) compared to DDRwt prostate cancer (Fig. 1c, d; Supplementary Data 3). CDK12d and MMRd samples exhibited a lower burden of copy number alterations and were more likely to have overall diploid genomes compared to the other classes (Fig. 1e, f; Supplementary Data 1). Prostate cancer is characterised in part by aneuploidy and structural rearrangements^21^ and in support of this, even the DDRwt samples showed a high burden of copy number variation and frequent evidence of high average ploidy (i.e. 3–4) suggestive of prior whole-genome doubling (Fig. 1f; Supplementary Fig. 1). Therefore, although BRCA2d samples were associated with increased ploidy and number of deletions relative to their base ploidy, there was high overlap in individual copy number features when compared to DDRwt samples (Fig. 1e, f; Supplementary Data 4, 5). Samples from patients with bladder cancer showed higher SNV burden compared to non-MMRd prostate cancer (with an enrichment of APOBEC-associated trinucleotide mutational signatures) and frequent aneuploidy (Fig. 1b–f) as previously reported^35^.

### Development of a machine learning classification model using whole-exome mutation and copy number features

**Fig. 2:**
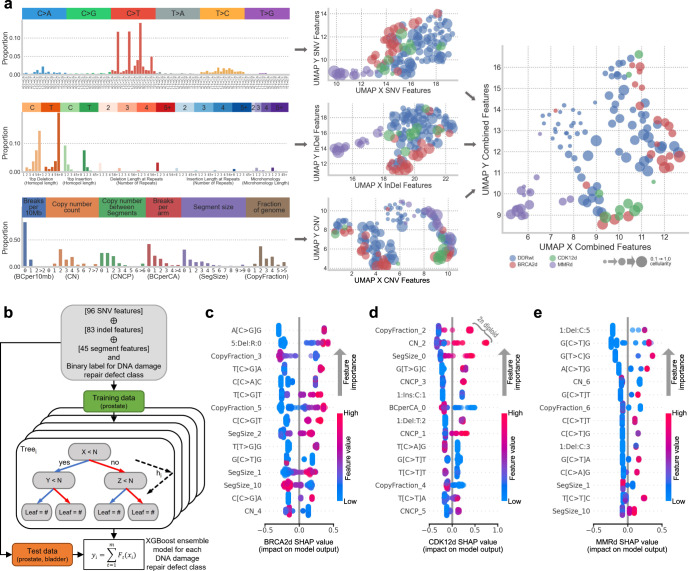
The impact of combined or individual genomic features on classification of DNA damage repair defects. **a** Left, The 224 total genomic features that formed the input for the machine learning classification model (top = single nucleotide variants (SNVs), middle = insertion deletion variants (InDels), bottom = copy number variants (CNVs)). See “Methods” for feature breakdown. Bars represent the average proportions across all prostate cancer samples. **a** middle, 2 dimensional UMAP representation of each feature category, coloured by DNA damage repair gene label. **a** right, UMAP representation of all feature categories horizontally concatenated together. **b** Schema of XGBoost model generation. All genomic features from samples with metastatic prostate cancer are horizontally concatenated as the model inputs, followed by a process of training many iterations to produce ensemble models for each class of DNA damage repair defect. The models were tested on both metastatic prostate and bladder cancer samples. **b**–**e** SHapley Additive exPlanations (SHAP) summary plots illustrating the 15 most impactful features for each classification model. Impact is determined as the sum of each feature’s sample level SHAP value. Feature values (blue to pink gradient) are normalised as a proportion of the highest value of each respective feature.

To develop a new classifier model we leveraged 224 individual somatic features (Supplementary Data 5, 6, 7), combining the established COSMIC SNV trinucleotide (*n* = 96) and InDel (*n* = 83) contexts^34^ with copy number segmentation features (*n* = 45) (Fig. 2a)^36^. In an unsupervised analysis using UMAP embeddings^37^, these somatic features showed promise for distinguishing MMRd samples and suggested that BRCA2d and CDK12d may be resolvable in a combined model (Fig. 2a). Samples with lower tumour fraction formed a distinct cluster (including a BRCA2d and CDK12d sample), presumably due to a reduction in the number of detected copy features.

The 224 somatic features and DDR labels for each sample served as the input for each eXtreme Gradient Boost (XGBoost) ensemble classifier (Fig. 2b; Supplementary Fig. 2), developing three separate binary models for BRCA2d, CDK12d and MMRd (collectively termed ‘DARC Sign’). Each model was trained on a schema of the respective DDR defect label versus all other samples, which included DDRwt and the two other defect labels. The samples in each class were split into test and training data and stratified on labels in a 40–60 split. A stratified K-fold cross-validation in a gridsearch of hyperparameters was then used to optimise a boosted decision tree ensemble model, and the main differentiating features of this model were interpreted using SHapley Additive exPlanations (SHAP) values (Supplementary Fig. 2; Supplementary Data 8, 9, 10, 11).

#### BRCA2

The canonical COSMIC signature 3 associated with HRR defects is relatively wide-ranging, including 34 different trinucleotide contexts (across all 6 substitutions classes) with proportions greater than 1%. Here, C-to-G base transversions (especially A[C > G]G) were the most impactful for BRCA2d classification, partly because other potentially relevant substitutions are prevalent in non-BRCA2d prostate cancers with ageing-related or MMRd-related etiologies (Fig. 2c; Supplementary Data 8; Supplementary Fig. 3). While deletions with microhomology at ligated DNA ends are frequently observed in BRCA2d genomes, they were not strongly impactful in our model (although InDels with a single micro-homologous base did contribute to the BRCA2d model). Instead, the number of long InDels was a stronger feature, likely because it is correlated with the number of indels with microhomology (Supplementary Fig. 4). As expected, copy number features that were representative of highly rearranged and aneuploid genomes were impactful for classifying BRCA2d (Fig. 2c; Supplementary Fig. 3). Consistent with approaches that leverage large-scale transitions to identify HRR deficiency, copy number segments of 10 and 20 Mb had a strong positive effect in the model while much larger segments (e.g. 100 Mb; SegSize_10: indicating a lower degree of copy number variation) contributed to a negative classification (Fig. 2c).

#### CDK12

Classification of CDK12d was driven by features associated with diploid genomes and focal copy number changes (Fig. 2d; Supplementary Data 9). With respect to the latter, enrichment of small segment sizes (below 5 kb) and absolute changes of just 1, 3, or 5 copies (i.e. features CNCP_1, CNCP_3, and CNCP_5, respectively) between adjacent segments were positively impactful—biologically representing consecutive tandem duplications of a single genomic tract—while an absence of breaks in a chromosome arm was a negative indicator of CDK12d (Supplementary Fig. 3). The copy number representation of short neighbouring segments with incremental copy changes in multiples of 2 is consistent with the established tandem duplicator phenotype of CDK12d cancers^5^.

#### MMRd

As expected, the MMRd model was strongly influenced by several mutational features that are rarely present in non-MMRd prostate cancer exomes (Fig. 2e; Supplementary Data 10). Genome-wide hypermutation is an established hallmark of MMRd tumours, resulting in an abundance of C > T mutations well beyond what can be causally attributed to ageing-associated spontaneous cytosine deamination (which also causes C > T mutations). Accordingly, N[C > T]G SNV features were highly impactful for MMRd classification. Features linked to microsatellite instability (another hallmark of MMRd) also strongly influenced classification: indeed, the most positively impactful feature was a 1 bp deletion in the context of a ≥ 5 bp cytosine homopolymer, uniquely attributed to replication slippage and unfaithful correction due to compromised mismatch repair (Supplementary Fig. 3). Although MMRd tumours (pan-cancer) are also typically associated with diploidy and a general absence of copy-number changes, features reflecting these properties are not exclusive to MMRd and therefore were comparatively less relevant in driving its classification (e.g. CDK12d genomes are also typically diploid).

### Model performance and exceptions

**Fig. 3:**
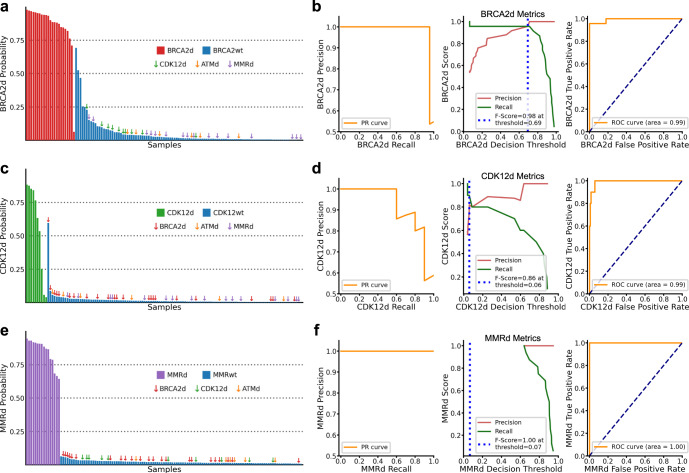
Performance of the DARC sign classification models. **a**, **c**, **e** Predicted probabilities of binary class membership for each of the three models, for all metastatic prostate cancer samples. **b**, **d**, **f** Associated classifier metrics for the performance of each model, based on the original labels. Measures include precision-recall curve (left), precision (red) and recall (blue) as function of decision threshold (centre) and ROC/AUC curves (right). Threshold (blue dotted line in centre panel) was determined as the minimum threshold to achieve the maximum F-score (see “Methods”).

For each model, we examined the probability of class membership based on the original sample labels (Fig. 3). Most samples with a BRCA2d label had a very high probability of BRCA2d class membership resulting in an F-score derived threshold of 0.69 and area under the curve (AUC) of 0.99 (Fig. 3a, b; Supplementary Fig. 5). Importantly, this included one BRCA2d sample with relatively low tumour fraction that had clustered separately in earlier unsupervised UMAP analysis. However, one sample (sample_011_PC) originally labelled as BRCA2d had a < 10% probability of BRCA2d class membership (Supplementary Data 12). While this sample carried a germline *BRCA2* stopgain mutation, the allele frequency of this mutation was suggestive of heterozygosity in the ctDNA sample and indicated that at least one intact copy of *BRCA2* remained present (Supplementary Fig. 6). Three samples that had not been previously identified in initial targeted sequencing had a high probability of BRCA2d class membership (including two samples from the same patient). Each exhibited characteristic InDel, SNV and copy features indicative of BRCA2d (Supplementary Fig. 6; Supplementary Data 5, 6, 7).

For CDK12d samples, a naive threshold of 0.5 results in three ‘false negatives’ (sample_036_PC, sample_037_PC and sample_039_PC; Fig. 3c; Supplementary Data 12). However, in two of three cases (sample_036_PC and sample_039_PC), another sample collected from the same patient (at a different time point) yielded a probability greater than 0.5. The ideal threshold likely falls above the F-score derived 0.09 but below 0.5 (Fig. 3d). One BRCA2d sample (sample_021_PC) showed a high probability of CDK12d class membership and exhibited some somatic features associated with CDK12d, including successive single copy changes and small copy number segments on chromosomes 3, 8, 9, 13 and 17 (Supplementary Fig. 7). However, whole-exome and targeted sequencing analysis revealed no *CDK12* gene alterations, and the overall widespread genomic instability of this sample was not indicative of CDK12d. Interestingly, CDK12d probability, but not BRCA2d or MMRd, was significantly associated with Sequenza-assessed tumour fraction (R = 0.26, *P* = 0.003 Wald Test) (Supplementary Fig. 8). This is likely because the classification of CDK12d is heavily reliant on copy number features that are difficult to identify in context of low tumour fraction^29^.

The MMRd subtype is a genomically-distinct outlier compared to non-MMRd samples. There are many singular features with strong discriminatory power for resolving MMRd from non-MMRd samples (e.g. 1:Del:C:5, A[C > T]G and C[C > T]G) (Supplementary Fig. 3). As such, our combined model incorporating all genomic features achieved perfect classification metrics with a broad threshold range between 0.06 and 0.64 (Fig. 3e, f; Supplementary Data 12).

### Utility in bladder cancer ctDNA whole-exomes

**Fig. 4:**
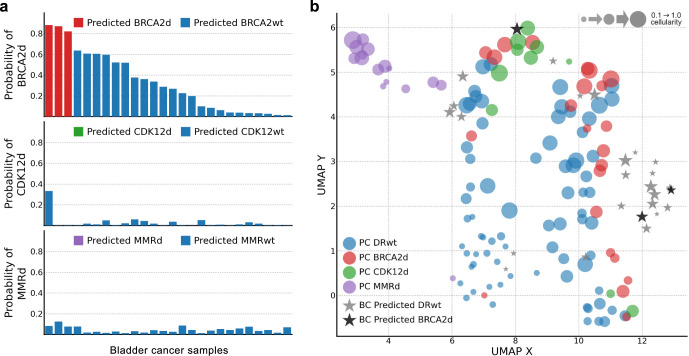
DARC sign models applied to metastatic bladder cancer ctDNA samples. **a** Predicted probabilities of class membership (using the models developed for metastatic prostate cancer) for 26 metastatic bladder cancer whole-exomes. All samples are column consistent between each row, and sorted by probability of BRCA2d class membership. **b** 2-dimensional UMAP representation including both metastatic prostate (circular points) and bladder samples (stars).

We applied our prostate cancer trained models to a cohort of 26 bladder cancer samples, which included one known case with deep *BRCA2* deletion (Supplementary Fig. 9). Three bladder cancer samples were classified as BRCA2d (Fig. 4a). The sample with a known *BRCA2* deep deletion had the highest BRCA2d probability (0.88). The two other samples (sample_145_BC and sample_151_BC) showed evidence of genomic alterations around the *BRCA2* locus, although with short-read WES we could not resolve any potential breakpoints (Supplementary Fig. 9). In a 2-dimensional UMAP with embeddings learned on all samples (prostate and bladder cancers), the original prostate cancer clusters are preserved while the majority of bladder cancer samples cluster apart, likely due to the increased number of APOBEC-associated SNVs (Fig. 4b). Probabilities for CDK12d and MMRd class membership were very low across all bladder cancer samples, consistent with the absence of these alterations in sporadic bladder cancer. Given the disparate SNV profiles for prostate and bladder cancer, these results provide evidence for the generalizability of our framework across other cancers.

### Comparison to an existing BRCA2d classifier

**Fig. 5:**
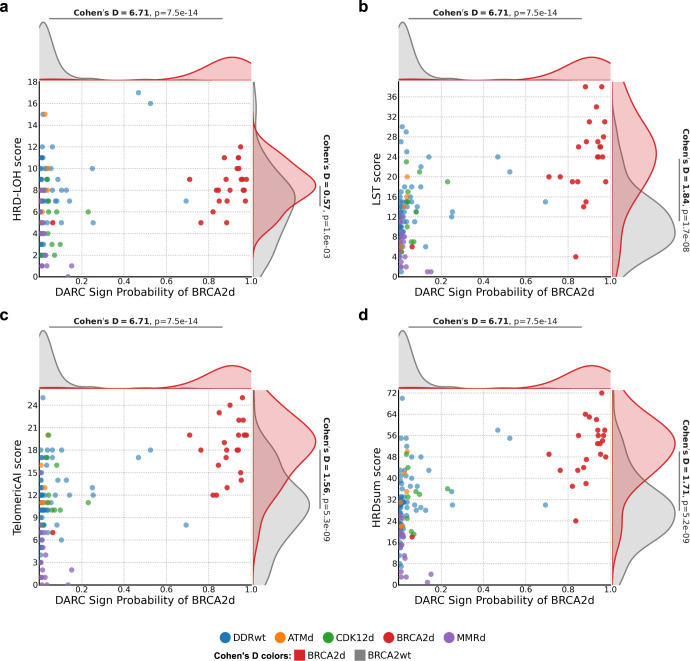
ScarHRD values compared to DARC sign BRCA2d probabilities. **a** Homologous Recombination Deficiency - Loss of Heterozygosity (HRD-LOH) score: the number of regions greater than 15 Mb (but not covering the entire chromosome) that show LOH. **b** Large Scale Transition (LST) score: the number of chromosomal breaks between adjacent regions of at least 10 Mb, with a distance between them not larger than 3 Mb. **c** Telomeric Allelic Imbalances (TelomericAI) score, the number of allelic imbalances that extend to the telomeric end of a chromosome. **d** Sum of scarHRD calculated values. *P*-value test statistics are from Mann–Whitney *U* tests. Cohen’s D is the standardised mean difference.

Most published tools including HRDetect and CHORD were designed for whole-genome sequencing data^15,23^ and are thus not appropriate comparisons to DARC Sign. We compared our model to scarHRD^38^ which uses WES to distinguish between *BRCA1/2* mutant and *BRCA1/2* wild type breast cancer. ScarHRD uses the sum of three copy number features: number of loss of heterozygosity (LOH) events, number of large scale transitions (LST) and number of copy number variants that extend to telomeric sequences (TelomericAI) (Fig. 5a–d; Supplementary Data 13). LOH and LST counts are also used as inputs in other machine learning-based classifiers^15,23^, and are being explored as standalone surrogates of PARP-inhibitor and/or platinum chemotherapy vulnerability (now reported as part of several commercial ctDNA assays)^25,39,40^. The number of LSTs (which is a feature called BCper10Mb in DARC Sign development) most strongly distinguished BRCA2d from BRCA2wt samples (Cohen’s d: 1.84, *p* = 3.4e-8), whereas number of LOH events (HRD score) was least discriminative for BRCA2d status (Cohen’s d: 0.57, *p* = 3.3e-3). Overall, DARC Sign achieved a much greater effect size and specificity for determining BRCA2d status (Cohen’s d: 6.71, *p* = 1.5e-13) than each naive copy number feature in isolation or as a sum (i.e. the output of ScarHRD). Collectively, these results highlight that individual copy number-based features from WES are insufficient to reliably predict *BRCA2* deficiency in metastatic prostate cancer. Rather, an approach combining multiple feature types provides superior discrimination, as shown here with DARC Sign.

## Discussion

We present DARC Sign, a framework for resolving multiple classes of clinically-actionable DDR deficiency in metastatic prostate cancer. Importantly, state-of-the-art machine learning models within DARC Sign use minimally-invasive liquid biopsy and standard WES data. In contrast, prior models for pan-cancer mutational signature derivation typically require costly whole-genomes and fresh frozen cancer tissue^15,23,24^.

PARP inhibitors are approved for mCRPC with selected HRR gene defects, but response rates are modest^2^. DARC Sign could feasibly be applied to large correlative efforts from clinical trials testing PARP inhibitors in prostate cancer (e.g. the Prostate Cancer Foundation supported PRECISION Registry; www.precision-registry.com) to clarify whether BRCA2d class membership may indicate particularly therapeutically-vulnerable cancers, including those with non-BRCA2-driven HRR deficiency. Rare mutations are reported in other HRR-related genes such as *PALB2* or *RAD51C* but the phenotypic consequences of these defects are unclear^12,41,42^. Importantly, prostate cancer poses a particular challenge for more naive single measure approaches that produce genomic instability scores, since copy number changes are pervasive (and therefore nonspecific to HRR-deficient genomes) and there are multiple classes of DDR defect associated with genome instability (including *CDK12* mutations) which are not associated with disease response to PARP inhibition^41^. It is notable that ‘HRD score’ thresholds developed for ovarian cancer have relatively poor accuracy in prostate cancer WES data^22^.

In contrast to previous whole-genome classifiers for HRR deficiency such as CHORD and HRDetect^15,23^, our models utilized additional genomic features and adjusted the weight of each feature based on the training data (rather than pre-selecting features). These additional features, including copy number alterations, likely compensated for the considerably reduced number of somatic alterations using our whole-exome approach. Prior signature-based research relying on WES mutational features alone show limited sensitivity and accuracy for identifying bona fide HRR-defective mCRPC^22,25^, partly due to the low mutational burden in prostate cancer. Nevertheless, the high specificity of our training data means that our model is likely best suited to metastatic prostate cancer, although the results from metastatic bladder cancer ctDNA indicate some potential for broader applicability. For other cancers, especially those with large differences in feature distribution relative to prostate cancer, it may be possible to train new model(s) using our open source framework, but this hypothesis will need to be tested with additional data. Re-training could also be performed for prostate cancer tumour tissue WES data, since it is plausible that the use of a ctDNA dataset introduced specificity due to more variable tumour fraction or greater sub-clonal diversity. A key strength of our study is the highly accurate labels for DDR gene status in ctDNA, which were derived from deep targeted sequencing with a bespoke prostate cancer panel, involved expert manual curation of both mutation and copy number data, and were validated in matched archival tissue^17,19^; most DDR gene testing is not as rigorous due to pragmatism in clinical settings. Future re-training efforts should aim to build a similarly accurate training dataset.

Our signature-based classifier demonstrated that it is possible to accurately resolve a CDK12d phenotype from HRR deficiency. Although *CDK12* mutations are associated with focal tandem duplications and can be described as genomically-unstable^5,6^, our model appeared to leverage the fact that CDK12d exomes were exclusively diploid and showed relatively few large copy number alterations. *CDK12* mutations are uncomplicated to identify with standard targeted sequencing and there do not appear to be complex or epigenetic events disrupting the *CDK12* locus in prostate cancer^17^. Nevertheless, there are likely to be cases with incidental *CDK12* mutations that do not exhibit a tandem duplicator phenotype. Since immunotherapy and CDK4/6 inhibition are currently in clinical trials for prostate cancer with *CDK12* mutations, there will be opportunities in future to determine the extent to which CDK12d classification is associated with disease response to various targeted therapies. A key limitation of our study is that we focused on samples with high ctDNA fraction, recognizing that additional sequencing depth (and therefore cost) would be required to recover somatic features from samples with low ctDNA fraction. However, even with high sequencing depth, copy number changes are difficult to detect in samples with low ctDNA fraction, while mutations and structural rearrangements can still be identified. The CDK12d classification model demonstrated the most reliance on copy number features and was expectedly therefore the most compromised by low ctDNA fraction. The potential utility of DARC Sign for identifying the CDK12d phenotype in ctDNA needs to be evaluated in context of this limitation.

Future studies should validate the generalizability of our classification tool to samples processed at other institutions (employing different laboratory and/or bioinformatic approaches), since a limitation of our study is a lack of external validation cohort (due to the relative rarity of publicly-available mCRPC cell-free DNA WES data). We also focused on samples with prior DDR gene alteration status defined by deep targeted sequencing and expert manual curation: necessary here for accurate training labels, but not always likely to be paired with WES or whole-genome sequencing in the long-term. In the near-term future, we suspect that even if broader sequencing approaches such as WES can inform on genomic signatures, targeted sequencing of ctDNA or tumour tissue is likely to retain clinical utility due to the importance of detecting genomic alterations outside of the DDR pathway, and also for identifying germline mutations or resistance mechanisms. Ultimately a theoretical strength of DARC Sign is the possibility to identify an HRR-deficient phenotype in the absence of a confirmed genomic alteration in a known HRR gene, including cancers with cryptic HRR gene alterations (e.g. intronic structural rearrangements or epigenetic silencing) that are difficult to detect with standard targeted DNA sequencing approaches. Conversely, DARC Sign is likely to separately classify cancers with an apparent HRR gene alteration but that retain functional HRR. Ideally, these theories should be tested in the context of mCRPC that is treated with a PARP inhibitor (or immunotherapy), where treatment responses can be correlated with both DDR gene alteration status and DARC Sign classification output. In our study we did not have sufficient numbers of cancers with *BRCA1*, *PALB2*, or other rare HRR gene alterations to determine whether DARC Sign would assign BRCA2d status to such cancers. Furthermore, we were not able to test whether some monoallelic HRR gene alterations are linked with DDR deficient phenotypes.

In conclusion, we developed a public machine learning tool (DARC Sign; DnA Repair Classification SIGNatures) that can identify and discriminate three different classes of clinically-relevant DDR defects in metastatic prostate cancer. Our models use WES data and minimally-invasive ctDNA rather than requiring expensive whole-genome sequencing and impractical fresh metastatic tissue biopsy. We believe that DDR signature-based classifiers leveraging both mutation and copy number based features will be useful for refining biomarker strategies.

## Methods

### Clinical cell-free DNA whole-exome sequencing cohort

We compiled WES data from our published cohorts of patients with clinically-progressing mCRPC accrued through clinical trials or a local biobank^19,28,43^. As part of these studies, all plasma cell-free DNA and patient-matched leukocyte DNA samples have undergone prior deep targeted sequencing, with a proportion also subjected to WES on the basis of high estimated ctDNA fraction^19,28,43^. To boost our sample size, we sequenced additional exomes from a cohort of DDR gene-altered mCRPC (again defined by prior targeted sequencing), prioritising samples with ctDNA fraction ≥20%^17^. Finally, we included published ctDNA WES data from 26 patients with metastatic bladder cancer as a comparator^32^. In total, 155 cell-free DNA samples with WES data passing quality controls were included (Supplementary Data 1). Although WES features of most samples within our cohort were partially characterised in prior studies^19,28,32,43^, the analyses undertaken here represent unique hypotheses exploring multiple classes of genomic signatures and applied machine-learning methods.

WES was performed on cell-free DNA and patient-matched leukocyte (germline) DNA using libraries previously prepared for targeted sequencing. Libraries were hybridised to the Roche NimbleGen SeqCap EZ MedExome capture panel according to the manufacturer’s protocols, and final enriched library pools were sequenced on Illumina machines^19,28,32^. Sequencing depths (Supplementary Data 2) were calculated using samtools mpileup (version 1.7)^44^ for all sites included in the EZ MedExome capture panel target files. Approval for collection and genomic profiling of patient samples was granted by the University of British Columbia Research Ethics Board. The study was conducted in accordance with the Declaration of Helsinki, and written informed consent was obtained from all participants prior to enrolment.

### Per-sample labels for DNA damage repair gene status

To construct a training dataset, all prostate samples were assigned a DDR gene status label. Sample labels reflect the presence or absence of putatively deleterious alterations in *BRCA2*, *ATM* and *CDK12*, determined from prior deep targeted exon sequencing of 22 DDR genes^17^. Mutation pathogenicity was assessed on the basis of predicted protein-level consequences: truncating variants—including frameshift InDels, splice site mutations (mutations within ±2 bp of an exon/intron junction), stop-loss, and nonsense (stop-gain) mutations—as well as deep deletions were considered deleterious. Germline and somatic missense mutations identified as ‘pathogenic’ or ‘likely pathogenic’ in the ClinVar database^45^ were also considered deleterious. Evidence of biallelic disruption was not required for a sample to be labelled as DDR gene defective, and samples without evidence of deleterious DDR gene defects were labelled as DDRwt (DNA damage repair wildtype). Labels of MMR genes (*MSH2/6*, *MLH1*) were previously defined using the same panel and criteria. In addition, clinical immunohistochemical staining of archival primary tissues and intron sequencing of *MSH2/6* and *MLH1* was previously performed to confirm MMRd gene status in selected samples^19^.

### Processing and alignment of whole-exome sequencing data

Paired-end reads were trimmed to remove adapters and bases with a quality score <20 were masked. Alignment to the GRCh38.p12 reference genome was performed using BWA-MEM (version 0.7.15)^46^. Samtools (version 1.7)^44^ was used for sorting and removal of reads with low quality mapping scores (MAPQ < 20). Duplicate reads were marked and removed with Picard MarkDuplicates (version 2.18.0) (http://broadinstitute.github.io/picard/). Pileup level statistics of base and InDel counts were generated using Pysamstats (https://github.com/alimanfoo/pysamstats).

### Somatic and germline variant calling

Somatic single nucleotide variants (SNVs) and InDels required a variant allele frequency (VAF) > 2% with at least 10 supporting unique reads at loci with >30× depth in both cell-free DNA and patient-matched leukocyte DNA. Candidate mutations were discarded if the VAF in cell-free DNA was less than 3× that of the paired leukocyte sample or less than 20× the background error rate, defined as the mean VAF of the position-matched substituted base(s) across all leukocyte samples. For variants adjacent to catalogued genomic repeats (via RepeatMasker) and/or regions with a strongly predominant base (i.e. the ±20 bp genomic context surrounding candidate variant being comprised of >80% a single nucleotide), we required a VAF > 40× the background error rate. Variants were also filtered if the mean distance from the end of supporting reads to the variant base was <6 bp. Functional annotation was performed using ANNOVAR^47^. Putative germline SNVs or InDels were identified from leukocyte samples and defined as non-reference bases with VAFs between 30% and 70% and a minimum read depth of 40×.

Whole-exome copy number variant calling (including local segmentation, determination of tumour purity and ploidy inference) was performed with Sequenza (version 3.0.0)^33^. We used default settings with a 10 kb window and min.reads.normal set to 20, and the result with the highest probability for each sample was accepted. For all samples in our study, non-diploid ploidies inferred by Sequenza (defined as mean ploidy <1.1 or >2.9) were only accepted if the Sequenza-determined cellularity was >0.18, since low tumour fraction typically precludes reliable determination of non-diploid status^29^.

### Feature generation

SigProfilerMatrixGenerator was used to create SNV trinucleotide contexts (*n* = 96) and InDel repeat context (*n* = 83) profiles^48^. No double base substitutions were found within our cohort and thus were not included as features. Established copy number features^36^ were calculated from the Sequenza segments output. The six copy feature categories include:

- BCper10mb: The number of 3’ segment endpoints per 10 Mb genomic window.
- BCperCA: The number of breaks per chromosome arm (as defined according to UCSC goldenPath hg38 database cytoBand.txt), calculated as the number of segments per arm minus one.
- CN: The number of segments with the associated integer copy number.
- CopyFraction: The fraction of the genome associated with each integer ploidy.
- SegSize: The segment length in units of 10 Mb.
- CNCP: The copy number change point is the absolute difference in ploidy between each segment and its 5’ neighbouring segment within the same chromosome.

### Modelling and downstream analysis

Separate datasets were generated for each label (BRCA2d, MMRd, CDK12d, DDRwt) such that for each dataset, samples with the respective label were marked as positive, and all other samples, including those with an alternate subtype label, were marked as negative. To generate cross-validated classification models for each label, samples were randomly assigned into testing and training sets using a 40:60 split stratified on the given label.

To determine the best scoring estimator and corresponding hyperparameters of XGBoost models for each label, a gridsearch K-fold cross-validation (sklearn.model_selection.GridSearchCV) was performed on each training dataset with 10 folds for the BRCA2d model and 6 folds for the CDK12d and MMRd models. The smaller k for the latter two models reflects the reduced availability of label-positive samples in these training sets due to the low population prevalence of CDK12d and MMRd (versus BRCA2d). A binary logistic objective was set for each model. Hyperparameters were scored based on an evaluation metric composed on the ordered criteria of AUC, error, and log loss. Through a maximum of one million rounds and a search space of 0.5–1.0 for the subsampling hyperparameters subsample, colsample_bytree, colsample_bylevel and colsample_bynode, an ensemble tree with a maximum depth of three nodes and a learning rate of 0.001 was found to be the highest scoring model for each of the three classifications. The resulting models fitted to each of the training datasets were used to predict the probability of the respective binary labels in the entire cohort including both prostate and bladder cancer samples.

For model interpretation, we analysed the impact of each feature on the ensemble models using the SHAP (SHapley Additive exPlanations) Python package (version 0.41.0)^49^. Shapley values were generated for each of the models using the “TreeExplainer” function with “feature_perturbation” set as “tree_path_dependent” and graphed in the style of the SHAP “summary_plot”. Feature importance was calculated as the sum of the absolute value of each sample’s feature attribution.

To assess model performance, AUC, receiver operating characteristics (ROC), F1-scores and other metrics were calculated using scikit-learn methods. F1-score-based thresholds were calculated by finding the F1 at all thresholds from 0 to 1 at 0.001 intervals, and the minimum threshold with the maximum F1 value was used as the derived threshold. UMAP values were generated using the umap-learn 0.4.3^37^ Python package using a Euclidean metric and 500 epochs. Trinucleotide mutation signature weights were calculated using DeconstructSigs (version 1.9.0) with default parameters^50^. All further downstream analysis including data processing and statistical tests were performed using Python 3.7, Pandas (version 1.4.2), NumPy (version 1.21.2), Scipy (version 1.7.3)^51^, and Scikit-learn^52^. To compare our ensemble boosted models to an established HRRd classifier, ScarHRD was run using default parameters, using the same Sequenza-generated inputs as used for DARC sign^38^.

### Reporting summary

Further information on research design is available in the Nature Research Reporting Summary linked to this article.

## Supplementary information

Supplementary Figures

Supplementary Data

REPORTING SUMMARY

## Data Availability

Unprocessed de-identified whole-exome sequencing reads for all cell-free and leukocyte DNA samples used in this study are available at EGA accession number EGAS00001007006 under standard controlled release. Previously published deep targeted exon-capture sequencing data for all samples analysed (plus select whole-exome sequencing data) are available at EGA accession numbers EGAS00001004800 (prostate cancer)^17^ and EGAS00001004615 (bladder cancer)^32^ under standard controlled release.

## Code availability

The DARC Sign models, code and structured data to reproduce results and figures in this study are public: https://github.com/elieritch/DarcSign. Code related to variant calling and annotation is publicly available at: https://github.com/elieritch/CircuiTT. All code provided depends on versions Python 3.7 or greater, Pandas 1.1 or greater, Seaborn 0.12 or greater, Scipy 1.7.3, Numpy 1.21.2, Scikit-learn 0.23.2, NumPy 1.21.2, umap-learn 0.4.3, SHAP 0.41.0 and XGBoost.
