# Supplementary material for: A generalizable machine learning framework for classifying DNA repair defects using ctDNA exomes
Source: NPJ Precis Oncol. 2023 Mar 13;7:27. doi: 10.1038/s41698-023-00366-z (PMC10011564; doi:10.1038/s41698-023-00366-z)
Supplement: Supplementary file 1 — Supplementary Figures [file 41698_2023_366_MOESM1_ESM.pdf]

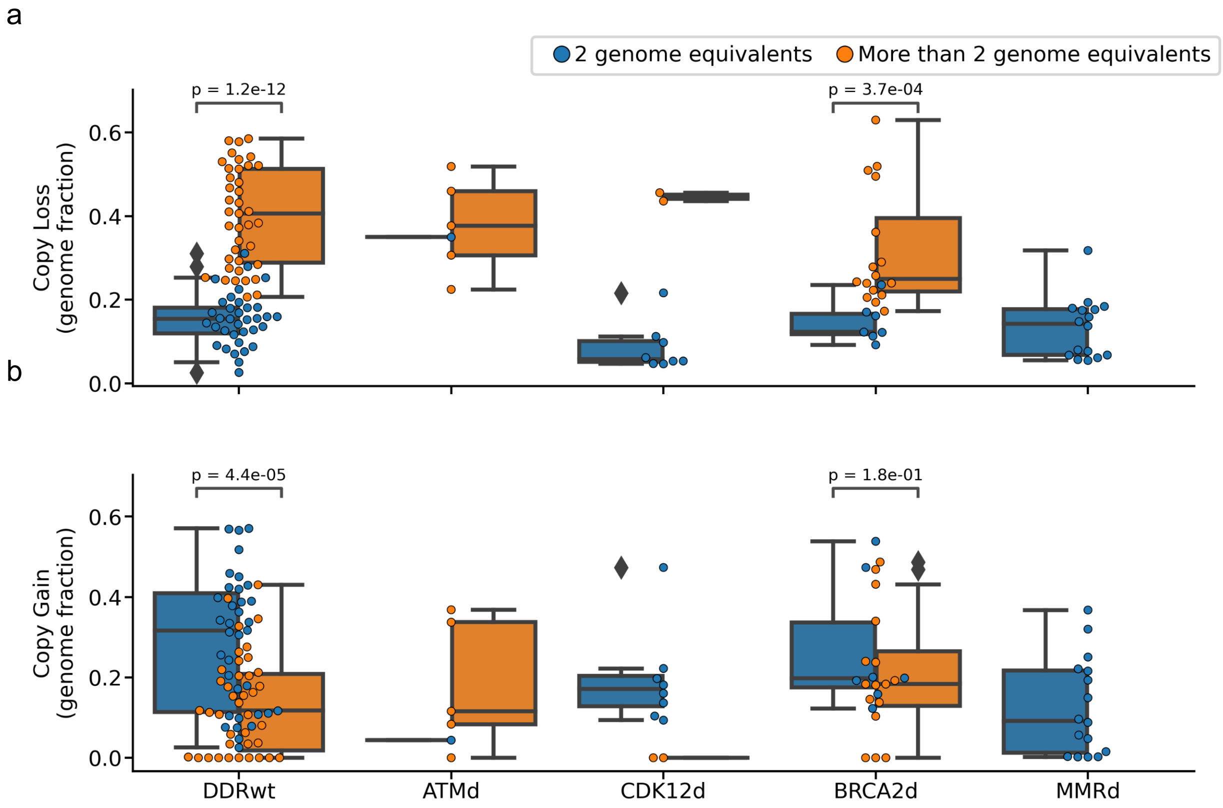

Supplementary Figure 1: Copy number gains and losses in the context of predicted whole genome doubling. Populations stratified by subtype label and Sequenza-estimated majority copy numbers less than or equal to 2 or greater than than 2 (*i.e.* representing a potential whole-genome doubling event). The Y axis represents the fraction of the genome with a copy number that is **a** less than or **b** greater than the majority copy number. P-values are from Mann–Whitney U tests.

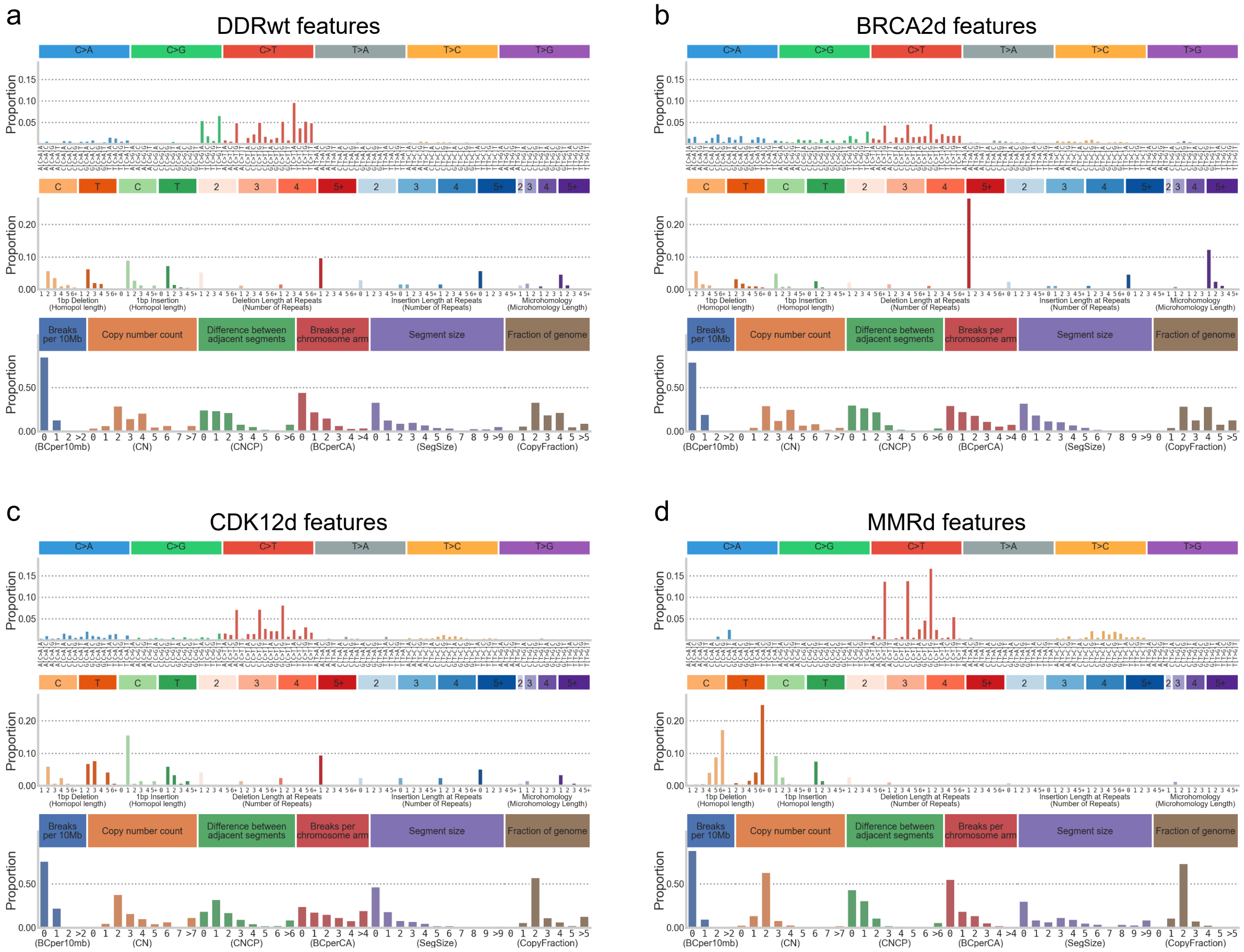

Supplementary Figure 2: Signature categories separated by subtype label. The mean proportion of COSMIC SNV trinucleotides contexts (96 features), indel contexts (83 features) and copy number segmentation features (45 features) for each subtype label highlights the differences between subtypes **a** DDRwt, **b** BRCA2d, **c** CDK12d, and **d** MMRd. While the model accepts raw counts as input, here average proportions in the total prostate cancer cohort are shown.

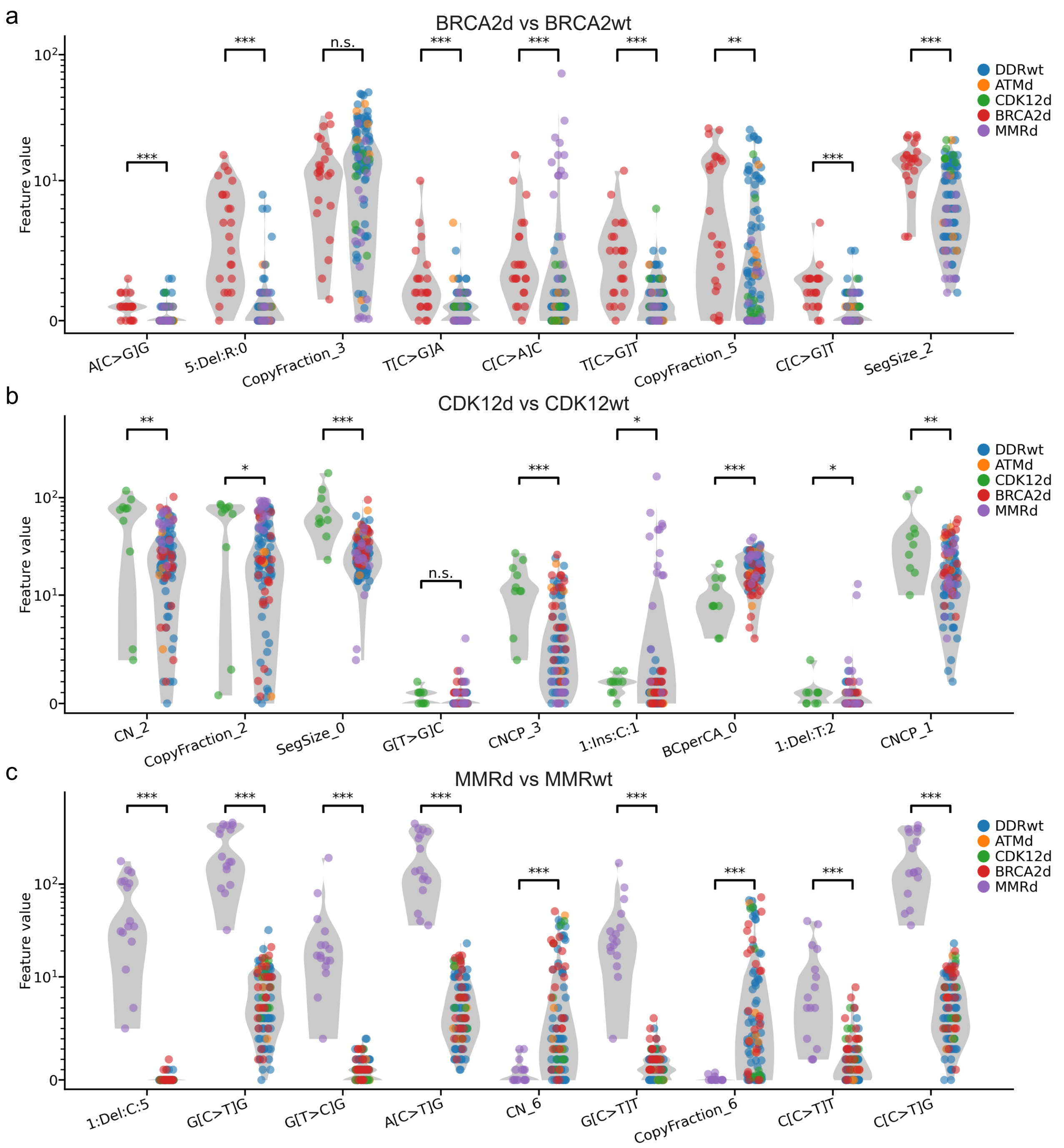

Supplementary Figure 3: Direct comparisons between features with the highest Shapely impact score. Nine features with the highest mean shapely impact, sorted by impact from left to right for each model including **a** BRCA2d, **b** CDK12d and **c** MMRd. Within-feature comparisons are between deficient (i.e. biomarker positive) versus proficient (i.e. negative controls) subtype status. Colours as per legends are consistent with prior figures. Statistical annotations are from two-sided Mann Whitney U tests and are annotated as n.s=P>0.05, *=P≤0.05, **=P≤0.01 and ***=P≤0.001.

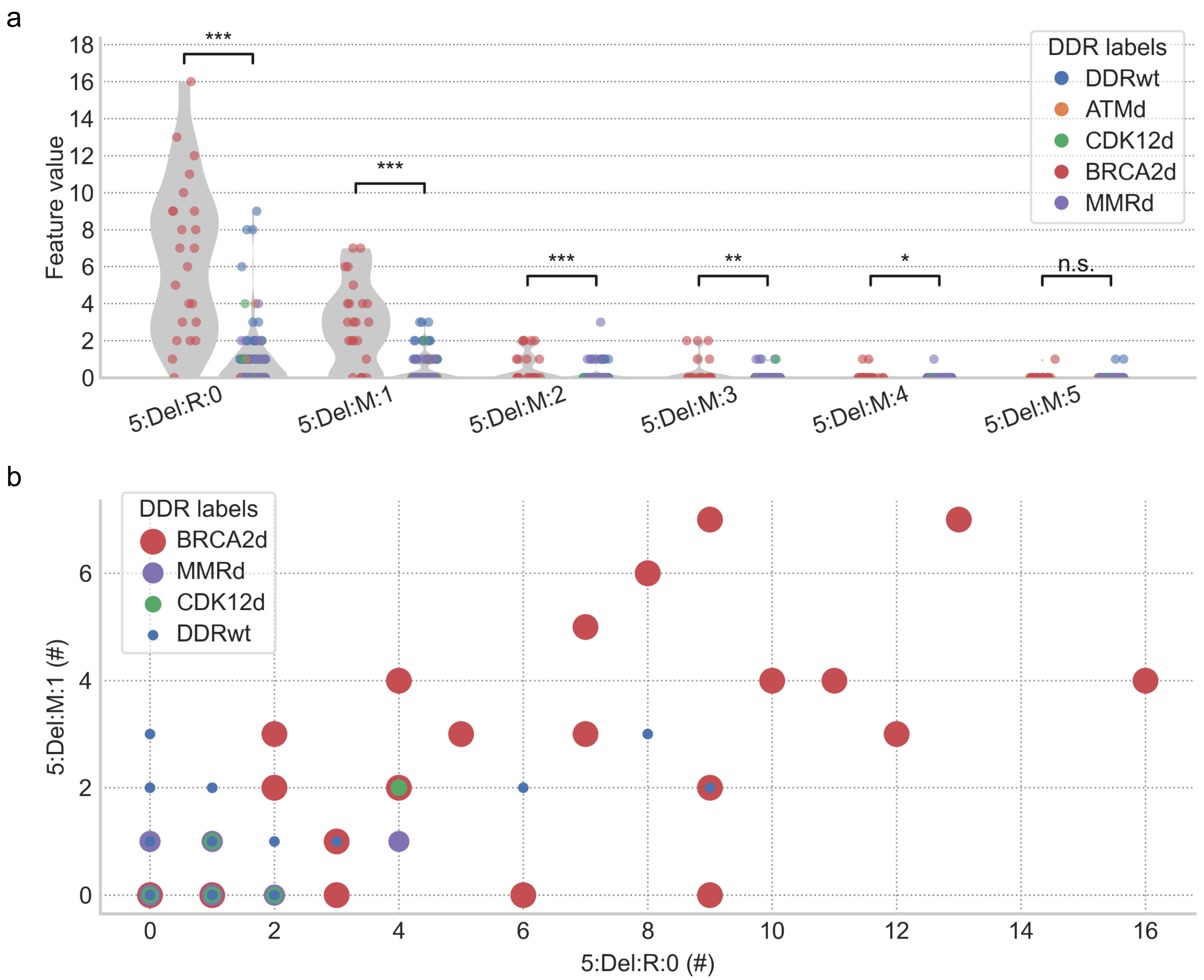

Supplementary Figure 4: Comparing the number of microhomologous indels and deletions greater than or equal to 5 bases. a The counts of selected InDel features in BRCA2d-labeled samples compared to all other samples. 5:Del:R:0 includes non-repetitive indels with greater than or equal to 5 bases, also included are comparisons of the microhomology categories. Statistical annotations are from two-sided Mann Whitney U tests. Statistical significances are annotated as n.s=P>0.05, *=P≤0.05, **=P≤0.01 and ***=P≤0.001. **b** The feature values of 5:Del:M:1 and 5:Del:R:0, stratified by DDR subtype label.

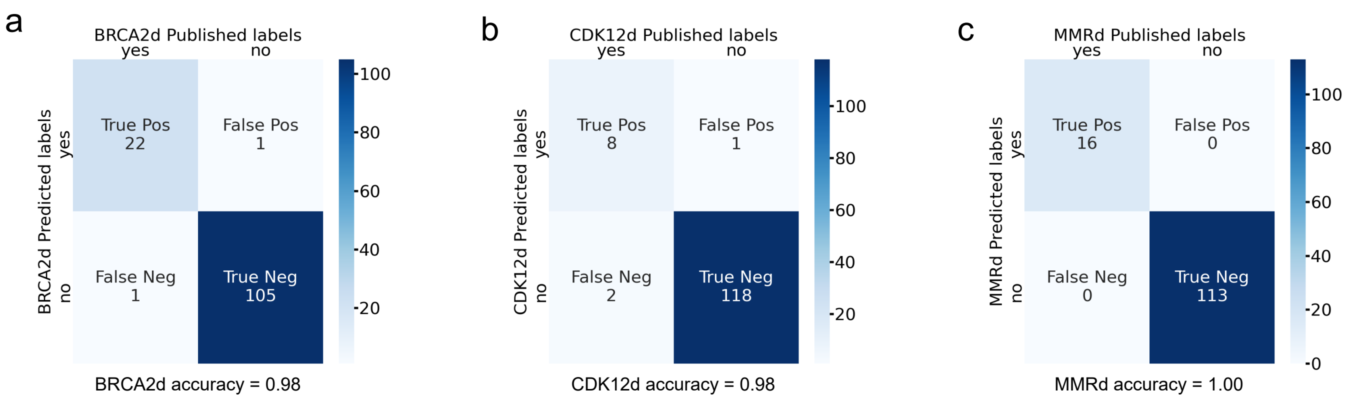

Supplementary Figure 5: Confusion matrices for BRCA2d, CDK12d, and MMRd models. Summary of the prediction results from each classifier model of **a** BRCA2d, **b** CDK12d, and **c** MMRd. Confusion matrix values are based on sample labels as previously described and the maximum f-score derived thresholds. Accuracies of each model are annotated below.

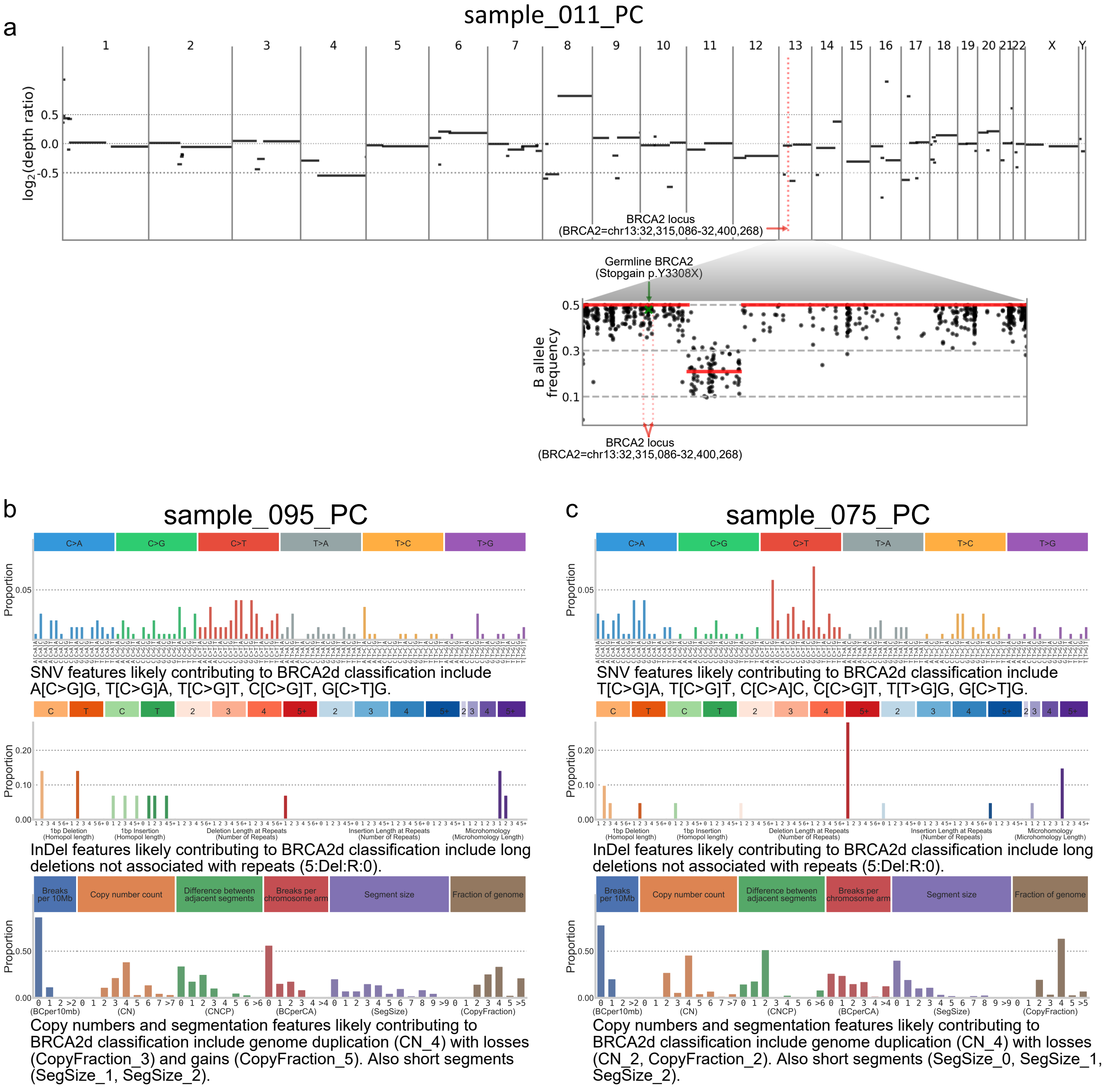

Supplementary Figure 6: Samples with disagreement between BRCA2d DARC Sign classification and gene panel-based labelling, with evidence supporting new classification. a A false negative sample (labelled BRCA2d-positive but classifier predicted BRCA2d-negative). Sequenza segmentation of genome log depth ratio (top). Fly out of B-allele frequency for chromosome 13 containing BRCA2 shows LOH in neighbouring BRCA2 locus (bottom). **b, c** False positive samples (labelled BRCA2d-negative but classifier predicted BRCA2d-positive). Signatures of samples displayed with captions highlighting impactful feature values that lead to each sample's classification.

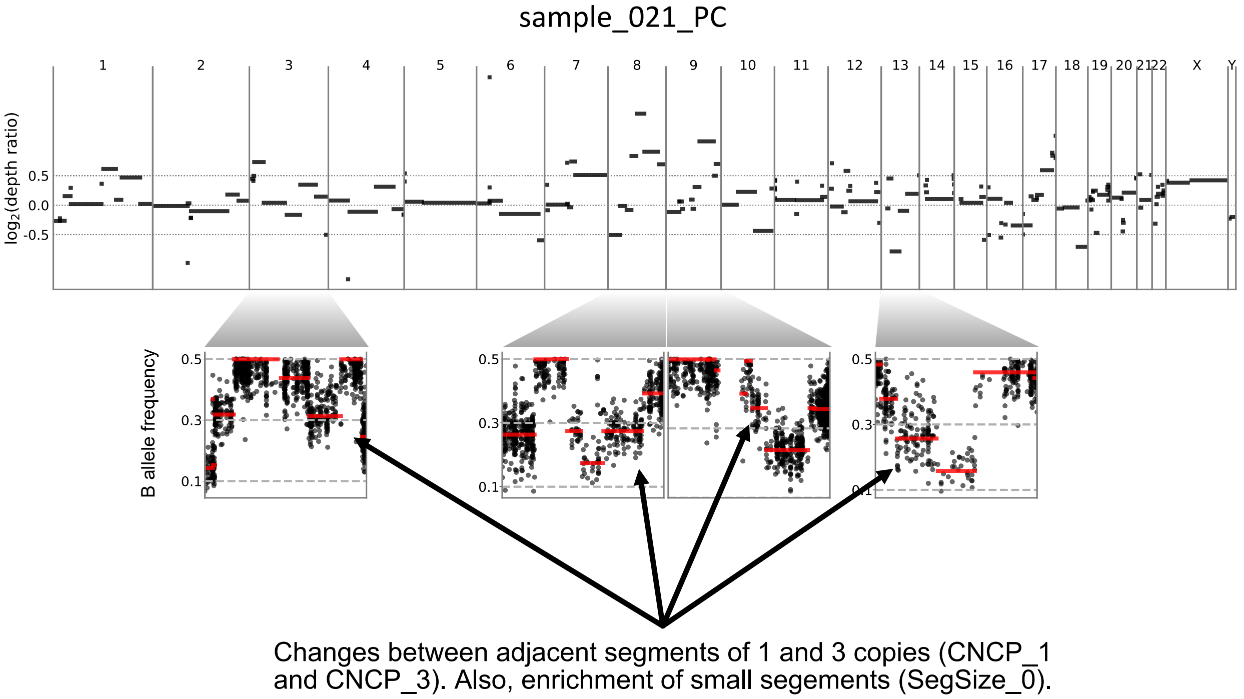

Supplementary Figure 7: Exome of suspected CDK12d instance. CDK12d False positive (labelled CDK12d-negative but classifier predicted CDK12d-positive) sample. Sequenza segmentation of coverage log depth ratios across the genome (top). B-allele frequencies as fly outs on chromosomes 3,8,9 and 13 (bottom) show regions demonstrating CDK12d features with high Shapely impact.

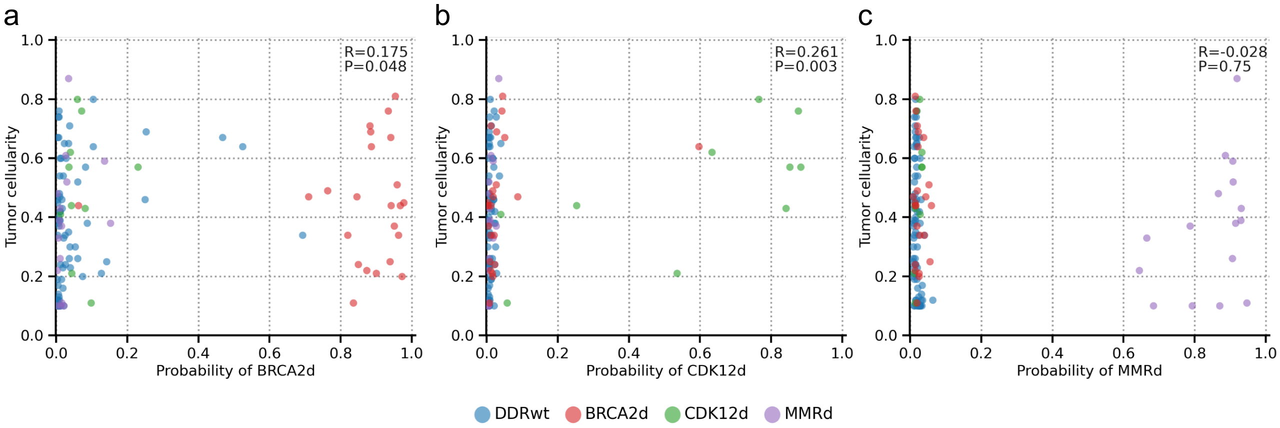

Supplementary Figure 8: The effect of tumour cellularity on each model. Tumour cellularity influences the ability to detect certain features. Its effects on sensitivity differ in each model depending on which features are the most impactful (e.g. copy-number versus mutation-based features). Displayed is the Sequenza-estimated tumour cellularity as a function of assigned probability of deficiency for **a** BRCA2d, **b** CDK12d, and **c** MMRd. Statistical annotations include R value correlation coefficients from linear least-squares regression of all data points and P value from two-sided Wald Test with t-distribution of the test statistic. Colours as per legends are consistent with prior figures.

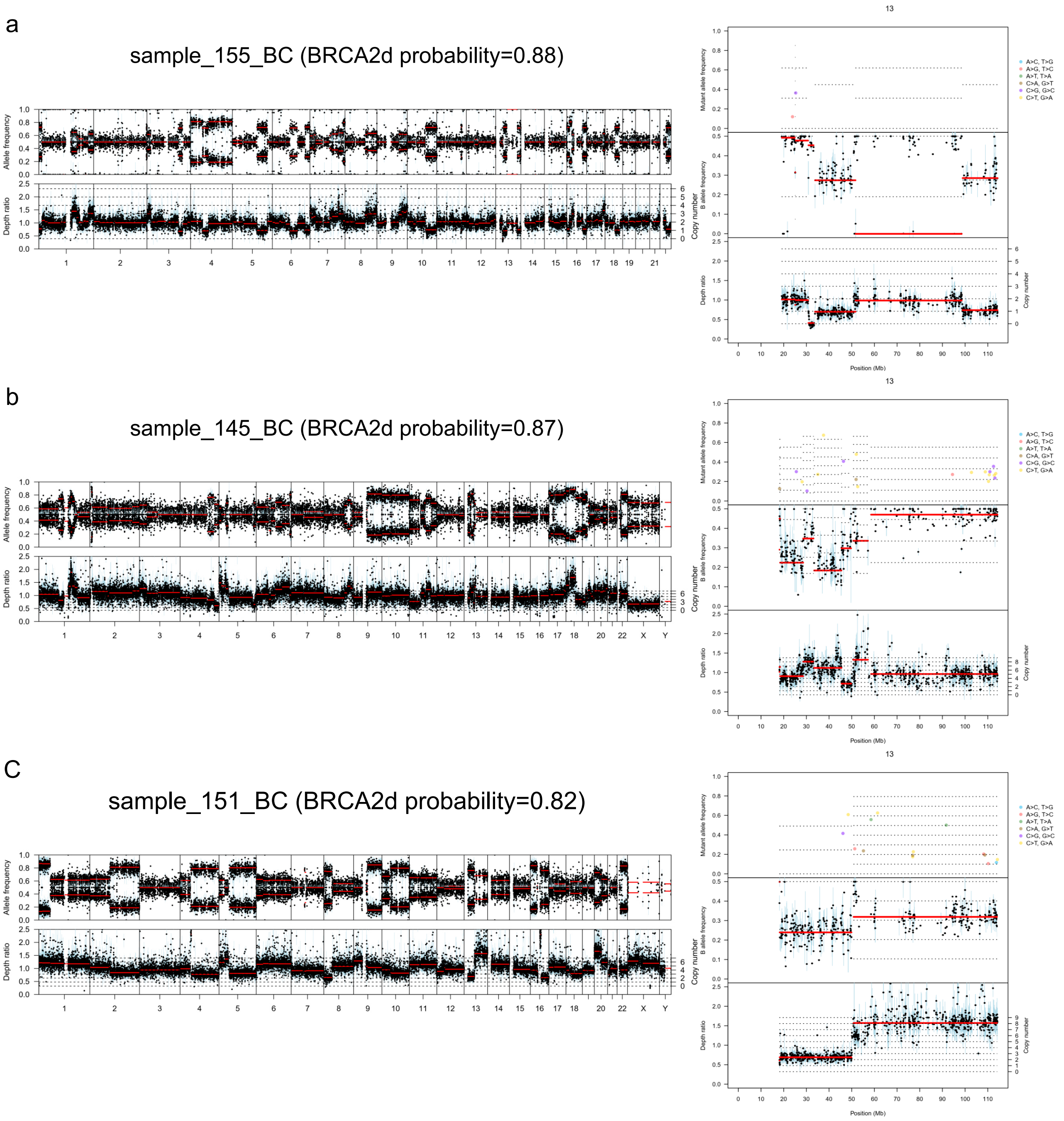

Supplementary Figure 9: Genomic profile of suspected BRCA2d metastatic bladder cancer. Genome-wide copy number profiles from Sequenza on the left and close-up view of chromosome 13 (including BRCA2) on the right. The BRCA2 gene includes genomic bins between 13:32316482-32398465. Probability of BRCA2d is annotated with sample names. **a** True positive sample that contains deep BRCA2 deletion identified with both targeted panel and whole-exome sequencing. **b, c** 2 additional samples with a classifier output of > 0.8 for BRCA2d.
